# Periodontally accelerated osteogenic orthodontics (PAOO) - a review

**DOI:** 10.4317/jced.50822

**Published:** 2012-12-01

**Authors:** Goyal Amit, Kalra JPS, Bhatiya Pankaj, Singla Suchinder, Bansal Parul

**Affiliations:** 1 MDS, Senior Lecturer. Department of Orthodontics and Dentofacial Orthopaedics, Guru Nanak Dev Dental College and Research Centre, Sunam, Punjab; 2 MDS, Professor. Department of Orthodontics and Dentofacial Orthopaedics, Guru Nanak Dev Dental College and Research Centre, Sunam, Punjab; 3 MDS, Associate Professor. Department of Orthodontics and Dentofacial Orthopaedics, Guru Nanak Dev Dental College and Research Centre, Sunam, Punjab

## Abstract

With an increasing number of adult patients coming to the orthodontic clinic, the orthodontic professional is constantly looking for ways to accelerate tooth movement. Surgical intervention to affect the alveolar housing and tooth movement has been described in various forms for over a hundred years. However, it is the spirit of interdisciplinary collaboration in orthodontics has expanded the realm of traditional orthodontic tooth movement protocols. Periodontal accelerated osteogenic orthodontics (PAOO) is a clinical procedure that combines selective alveolar corticotomy, particulate bone grafting, and the application of orthodontic forces. This procedure is theoretically based on the bone healing pattern known as the regional acceleratory phenomenon (RAP). PAOO results in an increase in alveolar bone width, shorter treatment time, increased post treatment stability, and decreased amount of apical root resorption. Tooth movement can be enhanced and cases completed with increased alveolar volume providing for a more intact periodontium, decreased need for extractions, degree of facial remodeling and increased bone support for teeth and overlying soft tissues, thereby augmenting gingival and facial esthetics.The purpose of this article is to describe the history, biology, clinical surgical procedures, indications, contraindications and possible complications of the PAOO procedure.

** Key words:**Periodontics, corticotomy, osteogenic, orthodontics.

## Introduction

An increasing number of adult patients are seeking orthodontic treatment (1). There are several psychological, biological and clinical differences between the orthodontic treatment of adults and adolescents. Adults have more specific objectives and concerns related to facial and dental aesthetics, the type of orthodontic appliance and the duration of treatment. Growth is an almost insignificant factor in adults compared to children, and there is increasing chance that hyalinization will occur during treatment (2). In addition, cell mobilization and conversion of collagen fibers is much slower in adults than in children. Finally, adult patients are more prone to periodontal complications since their teeth are confined in non-flexible alveolar bone (2).

These considerations make orthodontic treatment of adults different and challenging as well as necessitate special concepts and procedures, such as the use of invisible appliances, shorter periods of treatment, the use of lighter forces and more precise tooth movements. The development of corticotomy-assisted orthodontic treatment (CAOT) opened doors and offered solutions to many limitations in the orthodontic treatment of adults.

The new technique described here provides an increased net alveolar volume after orthodontic treatment. This is called the periodontally accelerated osteogenic orthodontics (PAOO) technique. It is a combination of a selective decortications facilitated orthodontic technique and alveolar augmentation (3). This method claims to have several advantages. These include a reduced treatment time, enhanced expansion, differential tooth movement, increased traction of impacted teeth and, finally, more post-orthodontic stability. With this technique, one is no longer at the mercy of the preexisting alveolar volume, and teeth can be moved 2 to 3 times further in 1/3rd to 1/4th the time required for traditional orthodontic therapy (3). The aim of this article is to present a comprehensive review of the literature, including the historical background, the contemporary clinical techniques, indications, contraindications, complications and side effects.

## Historical Review

Surgically assisted orthodontic tooth movement has been used since the 1800s. Corticotomy-facilitated tooth movement was first described by L.C. Bryan in 1893. However it was first introduced in 1959 by Kole (5) as a mean for rapid tooth movement. It was believed that the main resistance to tooth movement was the cortical plates of bone and by disrupting its continuity, orthodontics could be completed in much less time than normally expected. Kole’s procedure involves the reflection of full thickness flaps to expose buccal and lingual alveolar bone, followed by interdental cuts through the cortical bone and barely penetrating the medullary bone (corticotomy style). The subapical horizontal cuts connecting the interdental cuts were osteotomy style, penetrating the full thickness of the alveolus. Because of the invasive nature of Kole’s technique, it was never widely accepted.

Düker (6) used Kole’s basic technique on beagle dogs to investigate how rapid tooth movement with corticoto-my affects the vitality of the teeth and the marginal periodontium. The health of the periodontium was preserved by avoiding the marginal crest bone during corticotomy cuts. It was concluded that neither the pulp nor the periodontium was damaged following orthodontic tooth movement after corticotomy surgery. The results helped to substantiate the belief regarding the health of crestal bone in relation to the corticotomy cuts. Design of the subsequent techniques has taken this into consideration; the interdental cuts are always left at least 2 mm short of the alveolar crestal bone level.

A more recent surgical orthodontic therapy was introduced by Wilcko et al. (3,7) which included the innovative strategy of combining corticotomy surgery with alveolar grafting in a technique referred to as Accelerated Osteogenic Orthodontics (AOO) and more recently to as PAOO. Several reports indicated that this technique is safe, effective, extremely predictable, associated with less root resorption and reduced treatment time, and can reduce the need for orthognathic surgery in certain situations (3,4,8-12).

## Biology Underlying PAOO

In PAOO technique, cortical bone is scarred surgically on both labial and lingual sides of the teeth to be moved followed by grafting. The patient is seen every 2 weeks, and the rapid tooth movement produced after PAOO is substantially different than periodontal ligament cell-mediated tooth movement. Recent evidence suggests a localized osteoporosis state, as a part of a healing event called regional acceleratory phenomenon (RAP), may be responsible for the rapid tooth movement after PAOO (4).

RAP was first described by Frost (13) in 1983, although this phenomenon has been familiar to many histomor-phometrists since 1966. Frost (13) noted that the original injury somehow accelerated the normal regional healing processes. This acceleration is the regional acceleratory phenomenon. RAP usually occurs after a fracture, arthrodesis, osteotomy, or bone-grafting procedure, and may involve recruitment and activation of precursor cells necessary for wound healing concentrated at the site of injury (13,14). RAP is not a separate healing event, but it can expedite hard and soft-tissue healing stages two- to tenfold. Shih and Norrdin (15) demonstrated that when intraoral cortical bone was injured by corticotomy, RAP accelerated the normal regional healing processes by transient bursts of hard- and soft-tissue remodeling.

The two main features of RAP in bone healing include decreased regional bone density and accelerated bone turnover, which are believed to facilitate orthodontic tooth movement (4,16). Goldie and King (16) induced an osteoporosis state by depleting calcium intake in lactating rats and found an increase in the orthodontic tooth movement. In addition, reduced root resorption was demonstrated. Most recently, Wilcko et al. (3) showed radiographic evidence of an osteoporosis state in an alveolar bone treated with corticotomy, a characteristic seen in RAP. Additionally, the researchers found comparable tooth movement acceleration with small, round cortical perforations and with corticotomy cuts in a split-mouth design. This finding further supported that RAP is responsible for rapid orthodontic tooth movement.

The RAP begins within a few days of injury, typically peaks at 1–2 months, usually lasts 4 months in bone and may take 6 to more than 24 months to subside (3,4). As long as tooth movement continues, the RAP is pro-longed. When RAP dissipates, the osteopenia disappears and the radiographic image of normal spongiosa reap-pears. When orthodontic tooth movement is completed, an environment is created that favors alveolar re-mineralization.

## Case Selection

PAOO can be used to accelerate tooth movement in most of the cases requiring orthodontic treatment. It has been shown to be particularly effective in treating moderate to severe crowding, in Class II malocclusions requiring expansion or extractions, and mild Class III malocclusions (3,17).

PAOO can be used in both maxillary and mandibular arches. However, the decision regarding the site of PAOO can be made based on clinical judgement. For example, maxillary expansion generally requires more time than correction of mild mandibular anterior crowding. So a case with a narrow maxilla and mild anterior crowding may benefit with PAOO in the maxilla and traditional orthodontic therapy in the mandibular arch. On the other hand, a case of bimaxillary dentoalveolar protrusion requiring extractions in both the arches can be treated with PAOO to hasten the result in both the arches. Having both arches corrected in a similar time frame is ideal.

## Surgical Technique

The surgical technique for PAOO consists of 5 steps viz. raising of flap, decortication, particulate grafting, closure and orthodontic force application.

- Flap Design

A proper flap design is essential for the success of any surgical procedure. In PAOO also the flap should provide proper access to the alveolar bone wherein corticotomies are to be performed. Preservation of the gingival form is also important for proper esthetic appearance. The basic flap design is a combination of a full thickness flap in the most coronal aspect of the flap with a split-thickness dissection performed in the apical portions (18). The flap should be extended beyond the corticotomy sites mesially and distally so that vertical releasing incisions are not required. For esthetic purposes the papilla between the maxillary central incisors should be preserved on the labial and palatal aspects. Access to the labial alveolar bone in this area is achieved by “tunneling” from the distal aspect (18).

- Decortication

Decortication refers to the removal of the cortical portion of the alveolar bone. However, it should be just enough to initiate the RAP response and should not create movable bone segments. After flap elevation, decortications of bone adjacent to the malpositioned teeth is performed by using low-speed round burs under local anesthesia. In the PAOO procedure, decortication is performed at clinical sites without entering the cancellous bone, avoiding risk of damage to underlying structures, such as the maxillary sinus and the mandibular canal. The corticotomies may also be achieved with a piezoelectric knife (17,19). The corticotomies are placed on both the labial and lingual (palatal) aspects of the alveolar bone (18).

- Particulate Grafting

The materials most commonly used for grafting after decortication are deproteinized bovine bone, autogenous bone, decalcified freeze-dried bone allograft, or a combination thereof (18). Grafting is done in most areas that have undergone corticotomies. The volume of the graft material used is dictated by the direction and amount of tooth movement predicted, the pretreatment thickness of the alveolar bone, and the need for labial support by the alveolar bone. A typical volume used is 0.25 to 0.5 ml of graft material per tooth (18).

- Closure Techniques

The flap should be closed using non resorbable interrupted sutures without creating excessive tension. No packing is required. The sutures are usually left in place for 1 to 2 weeks (18).

- Timing of Orthodontic Treatment

The placement of orthodontic brackets and activation of the arch wires are typically done the week before the surgical aspect of PAOO is performed. However, if complex mucogingival procedures are combined with the PAOO surgery, the lack of fixed orthodontic appliances may enable easier flap manipulation and suturing. After flap repositioning, an immediate heavy orthodontic force can be applied to the teeth and in all cases initiation of orthodontic force should not be delayed more than 2 weeks after surgery. A longer delay will fail to take full advantage of the limited time period that the RAP is occurring. The orthodontist has a limited amount of time to accomplish accelerated tooth movement. This period is usually 4 to 6 months, after which finishing movements occur with a normal speed (18). Given this limited “window” of rapid movement, the orthodontist will need to advance arch wire sizes rapidly, initially engaging the largest arch wire possible.

## Indications and Clinical Applications

Several clinical applications for PAOO have been reported (3,9,10,19,20,21,22). Corticotomy was used to facilitate orthodontic tooth movement and to overcome some shortcomings of conventional orthodontic treatment, such as the long required duration, limited envelope of tooth movement and difficulty of producing movements in certain directions. These applications include the following:

1. Resolve Crowding and Shorten Treatment Time

2. Accelerate Canine Retraction after Premolar Extraction

3. Enhance Post-Orthodontic Stability

4. Facilitate Eruption of Impacted Teeth

5. Facilitate Slow Orthodontic Expansion

6. Molar Intrusion and Open Bite Correction

7. Manipulation of Anchorage

## Contraindications and limitations

Patients with active periodontal disease or gingival recession are not good candidates for PAOO. In addition, PAOO should not be considered as an alternative for surgically assisted palatal expansion in the treatment of severe posterior cross-bite. PAOO also should not be used in cases where bimaxillary protrusion is accompanied with a gummy smile, which might benefit more from segmental osteotomy (10).

## Complications and Side Effects

Although PAOO may be considered a less-invasive procedure than osteotomy-assisted orthodontics or surgically assisted rapid expansion, there have still been several reports regarding adverse effects to the periodontium after corticotomy, ranging from no problems to slight interdental bone loss and loss of attached gingiva, to periodontal defects observed in some cases with short interdental distance (7,22-24). Subcutaneous hematomas of the face and the neck have been reported after intensive corticotomies (8,25). In addition, some post-operative swelling and pain is expected for several days.

No effect on the vitality of the pulps of the teeth in the area of corticotomy was reported (8). Long-term research on pulpal vitality after rapid movement has not been evaluated in the literature. In an animal study, Liou et al. (26) demonstrated normal pulp vitality after rapid tooth movement at a rate of 1.2 mm per week. However, pulp vitality deserves additional investigation.

It is generally accepted that some root resorption is expected with any orthodontic tooth movement (27). An association between increased root resorption and duration of the applied force was reported (28-30). The re-duced treatment duration of PAOO may reduce the risk of root resorption. Ren et al. (31) reported rapid tooth movement after corticotomy in beagles without any associated root resorption or irreversible pulp injury. Moon et al. (32) reported safe and sufficient maxillary molar intrusion (3.0 mm intrusion in two months) using corticotomy combined with a skeletal anchorage system with no root resorption. Long-term effect of PAOO on root resorption requires further study.

## Discussion

The fact that the teeth can be moved more rapidly, thus resulting in shortened treatment times, is certainly advantageous to the patient’s periodontal health because less time in fixed appliances reduces patient “burnout” and substantially reduces the time available for relatively benign commensal bacterial biofilms to assume qualitative changes and convert to a destructive cytotoxic potential often seen when fixed appliances have remained on the teeth for more than 2 to 3 years. The significance of the increase of the rate of tooth movement, however, pales in comparison to the fact that the teeth can be moved two to three times further than would be possible with traditional orthodontics alone, and that the cases can be completed with an increased alveolar bone volume (21). This increased alveolar volume can provide for a more intact periodontium, a decreased need for extractions, a degree of facial reshaping, and an increase in the bony support for both the teeth and the overlying and soft tissues.

The ability to increase the post treatment alveolar volume and cover vital root surfaces can result in the repair of preexisting alveolar dehiscences over root prominences and lessen the likelihood of new dehiscence formation, which can be a contributing factor to gingival recession (25).

## Conclusion

From an esthetic perspective the PAOO technique not only addresses tooth alignment, but also facial features and, as such, is truly in vivo tissue engineering. With a combination of both in-office periodontal surgery and orthodontic treatment, we can now more routinely address the esthetics of the entire lower face. The PAOO technique requires the utilization of numerous modified diagnostic and treatment parameters, but once these are mastered the orthodontist has a powerful new treatment option to offer his or her patients. With the increasing number of adults considering orthodontic treatment, the propensity for adults and even some non growing adolescents for periodontal problems, the PAOO technique can be an especially attractive treatment option and be a “win-win” situation for the orthodontist, the periodontist and the patient.
